# Monthly Summary

**Published:** 1867-08

**Authors:** 


### MONTHLY SUMMARY.

---

*Heat produced by Mental work.*—Dr. Lombard, assistant professor of Physiology in Harvard University, has published a paper in a recent number of the New York Medical Journal, in which he attempts to show that mental work increases the heat of the head.

For the purpose of his inquiries, he employed a thermo-electric apparatus capable of indicating exceedingly minute variations of temperature, less than a thousandth part of a centigrade degree. He chose himself as the subject of his experiments which extended over the space of a year. His first effort was to determine the normal temperature of his head, when at rest from all mental work. In this he found much variation, the temperature being sometimes steady, at others rising and falling, often with considerable rapidity. Inquiry into the source of these variations satisfied him that when the temperature was unchanged his mind was in a torpid condition, while in the other case, it was more or less occupied. Attention to conversation was sufficient to increase the heat of the head. It was evident that no augmented action of the heart could account for this phenomenon, as the temperature of other parts of the body more favorably situate for feeling such influence than the head, remained unchanged.

Exercise of the reasoning powers heated the head to the extreme extent of one twentieth of a degree of the centigrade thermometer, and this elevation was accompanied by a depression of the temperature of other parts of the body. The emotions, however, were found to be more powerful excitors of the brain. Reading poetry which roused the interest and excited the sympathies, was the most active of all the means he experimented on, the temperature being more elevated by a few minutes recitation than by hours of deep thought.

The statement of these results shows the author to be rather unfortunate in his phraseology. Strictly speaking this is not *mental work* but *emotional excitement.* The tendency of blood to the head during strong emotion is well known. In this same number we find some illustrations of this fact. Dr. Howe of Philadelphia, reports a case of fracture of the skull, in which the operation of trepanning exposed a considerable portion of the brain. Observations on this showed that during sleep, the membranes did not protrude so far as while the boy was awake, and that the pulsations of the brain were more feeble, indicating a lower vascular activity. The more active and energetic the emotions, the greater the protrusion of the brain. On several occasions, when the child was frightened or angry, the parts were much pushed out, and sometimes the distension was so great as to produce slight hemorrhage.

---

*Gases in Meteoric Iron.*—Prof. Graham of the English Mint has investigated the gases contained in meteoric iron, and has reached some remarkable conclusions. He finds that a pure malleable meteoric iron, of specific gravity 7.79, gives off when heated in a vacuum for 2½ hours, 2.85 times its volume of gas, composed as follows:

| | |
|---|---|
| Hydrogen | 85.68 |
| Carbonic Oxide | 4.46 |
| Nitrogen | 9.86 |
| | 100.00 |

It contained not a trace of carbonic acid, nor any hydrocarbon vapour absorbable by sulphuric acid.

Now, as it is known that iron is capable of absorbing or including gases from an ordinary fire, Graham made a comparative test, selecting clean horse-shoe nails as the substance to be operated upon. He found that, when similarly heated for 4½ hours, they gave off 2.66 times their volume of gas having the following composition.

| | |
|---|---|
| Hydrogen | 35.0 |
| Carbonic Oxide | 50.3 |
| Carbonic Acid | 7.7 |
| Nitrogen | 7.0 |
| | 100.0 |

The difference in the constitution of these gases is very marked. It consists chiefly in the great excess of hydrogen, the small proportion of carbonic oxide, and the absence of carbonic acid in the air expelled from meteoric iron. It reveals a totally different constitution of the atmosphere in the remote regions from which it has come. It is no easy matter to impregnate iron in our atmosphere with its own volume of hydrogen, but this meteoric iron gave up three times that quantity without being fully exhausted. It must therefore have been fused in a dense atmosphere of hydrogen gas. Now the spectroscope reveals to us the presence of hydrogen in large quantity in the fixed stars, so that we may regard this hydrogen as imported to us from starry realms far beyond the limits of the solar system.

---

*New Treatment of Necrosis.*—A letter from London to the *Richmond Journal* informs us that a new treatment of necrosis has been introduced by Sir William Ferguson. Ordinarily the bone is let alone till the dead is separated from the living portion, after which it is removed. By this plan, the sequestrum is often allowed to be nearly completely enveloped by new bone. Sir William Ferguson, on the contrary, as soon as necrosis is detected, cuts down at once upon the diseased bone, dividing the periosteum. This prevents the formation of new bone under the severed periosteum, and facilitates the removal of the dead portion.

---

*Hydrophobia.*—Professor St. Cyr, of the Lyons Veterinary School, corrects some popular errors touching this frightful disease. He tells us:

1. The disease in dogs has no definite period of incubation, which varies from 16 to 115 days, and the short period of 2 or 3 days, spoken of in rural districts has no foundation in fact.
2. Bitches are as liable to hydrophobia as dogs.
3. Dogs are more liable in proportion to the wandering character of their lives.
4. Hot and dry weather does not favour the occurrence of canine madness, the rainy months producing more cases of the disease.
5. No cause but contagion is known.

The period of incubation in man is known to vary. A case has recently been reported in Liege, in which the time of the poison was clearly ascertained. The man was attacked with hydrophobia one year and six days after the application of the rabid animal's saliva.

---

*New Base for Artificial Teeth.*—Dr. G. F. J. Colburn, of Newark, N. J., has invented a substitute for rubber in dentistry, which promises to be of much value to the profession. It is in reality a cement of which the mineral asbestos is one of the ingredients. Asbestos is a very peculiar substance. It it excedingly light, and so very fibrous in its nature that it may be spun and woven like cloth, in which condition it resists fire, water and many of the acids with complete success. Taking advantage of these natural qualities Dr. Colburn has by long study, discovered additional substances, which, when united, form an artificial base that posesses remarkable toughness, adherence, strength and lightness. The ease and freedom with which it can be molded is a strong recommendation. It can be readily applied to gold, platinum and other plates. We have seen some full sets of teeth on aluminum plates that were truly beautiful. This new base contains no ingredients injurious to the health of the mouth or system. It is not affected by acid secretions, is free from all taste, and is inodorous. We hope that its merit, will be thoroughly tested. Patents have been allowed.

---

### EDITORIAL DEPARTMENT.

